# Long-Lasting Effects of Maternal Condition in Free-Ranging Cervids

**DOI:** 10.1371/journal.pone.0058373

**Published:** 2013-03-05

**Authors:** Eric D. Freeman, Randy T. Larsen, Ken Clegg, Brock R. McMillan

**Affiliations:** 1 Brigham Young University, Plant and Wildlife Sciences Department, Provo, Utah, United States of America; 2 Private Lands Consulting, Springville, Utah, United States of America; Université de Sherbrooke, Canada

## Abstract

Causes of phenotypic variation are fundamental to evolutionary ecology because they influence the traits acted upon by natural selection. One such cause of phenotypic variation is a maternal effect, which is the influence of the environment experienced by a female (and her corresponding phenotype) on the phenotype of her offspring (independent of the offspring’s genotype). While maternal effects are well documented, the longevity and fitness impact of these effects remains unclear because it is difficult to follow free-living individuals through their reproductive lifetimes. For long-lived species, it has been suggested that maternal effects are masked by environmental variables acting on offspring in years following the period of dependence. Our objective was to use indirect measures of maternal condition to determine if maternal effects have long-lasting influences on male offspring in two species of cervid. Because antlers are sexually selected, we used measures of antler size at time of death, 1.5–21.5 years after gestation to investigate maternal effects. We quantified antler size of 11,000 male elk and mule deer born throughout the intermountain western US (6 states) over nearly 30 years. Maternal condition during development was estimated indirectly using a suite of abiotic variables known to influence condition of cervids (i.e., winter severity, spring and summer temperature, and spring and summer precipitation). Antler size of male cervids was significantly associated with our indirect measure of maternal condition during gestation and lactation. Assuming the correctness of our indirect measure, our findings demonstrate that antler size is a sexually selected trait that is influenced–into adulthood–by maternal condition. This link emphasizes the importance of considering inherited environmental effects when interpreting population dynamics or examining reproductive success of long-lived organisms.

## Introduction

Causes of phenotypic variation are fundamental to evolutionary ecology because they influence the traits acted upon by natural selection [1]. A particular phenotype (among many in a population) may be the result of an individual’s genotype, environment, or an interaction between the two [2]. More specifically, the environment can affect an individual’s phenotype through experiences during development, or through “inherited environmental effects” [3], [4]. Commonly, these inherited environmental effects are the impact of a mother’s phenotype on her offspring, often a result of the environment experienced by the mother [5]. These effects on offspring phenotype are known as maternal effects.

By definition, maternal effects are “the influence of the maternally provided environment on the phenotype of her offspring” [6]. Often, maternal effects occur when an offspring’s phenotype is influenced by the environment experienced by its mother while *in utero* or during dependency [3], [7]. These maternal effects may have a positive or negative influence on the fitness potential of offspring [8], [9]. This influence occurs as females balance the allocation of resources between maintenance functions, current-year reproduction, and future reproduction [10]. When resources are abundant, females may allocate additional energy toward the current-years reproduction, thereby producing higher quality (larger, heavier, etc.) offspring [11], [12]. However, when resources are limited, mothers may invest less in their offspring so as to maintain their own health and ensure their future reproductive potential [13]. In both cases, a maternal effect may be passed on to the subsequent generation, potentially affecting offspring size, growth, survival, reproductive success, or other demographic parameters [14].

While maternal effects are well documented, the longevity and impact of these effects remains unclear as most available data evaluate maternal effects only through the first 1–4 years of life. For example, Schultz and Johnson found maternal effects influenced body mass of white-tailed deer (*Odocoileus virginianus*) through 2.5 years [15]. Other examples include studies of bison (*Bison bison*; 4 years of maternal effects) [16] and reindeer (*Rangifer tarandus*; 18 months) [17]. This lack of information likely occurs because it is often logistically difficult to collect evidence that maternal effects have long-lasting influences on life-history characters or the potential reproductive success of offspring, especially for long-lived species (i.e. male offspring disperse relatively large distances and their reproductive success is therefore difficult to assess). In contrast, observations of bighorn sheep in Canada (*Ovis canadensis*; body mass at 5 years was influenced by maternal effects) [18] and red deer *(Cervus elaphus)* on the Isle of Rum (Scotland; birth weight and the dominance of hinds was correlated with the lifetime reproductive success of male offspring) have offered a thorough examination of the impacts of maternal effects [19], [20]. Other studies of maternal effects in long-lived species (e.g., ungulates) often fail to consider the entire reproductive lifetime of individuals [21]. Additional difficulties include collecting a large sample from across the range of the species. Robust data (temporally and spatially) are likely important when determining what impact maternal effects may have on the life history of a species. Moreover, Hewison and Gaillard proposed that, “because male ungulates do not usually breed until several years after maternal investment has ceased … environmental factors might mask any maternal influence on reproductive success of sons”, further complicating assessment of the influence of maternal effects (pp. 233) [21].

Ungulate populations may provide a model system to study maternal effects because individuals are relatively long-lived and short-term maternal effects are well established [22], [23], [24]. For example, the offspring of older female moose (*Alces alces*) generally have greater birth masses and survival rates through their first summer [25]. This model system is facilitated by the relatively easy identification of female-offspring relationships in these generally large, terrestrial species [5]. For example, Jones et al. [26] demonstrated that after accounting for maternal mass and size, a female’s age does not significantly affect the survival of her offspring, yet the offspring of yearling Soay sheep (*Ovis aries*) mothers exhibit lighter birth masses and lower survival. This association illustrates that the lower survival of these offspring is likely not a result of lesser maternal experience, but is instead a result of decreased maternal condition in yearling mothers. Conversely, the survival of young white-tailed deer increases with maternal age (likely related to both condition and experience) [27]. In several similar studies, the offspring of heavier mothers have greater survival [10] or birth masses [28].

The limitation of using ungulates (or any other long-lived species) to assess the influence of maternal effects on offspring is that it is difficult to follow free-living individuals and obtain measures of condition or quantify life history characters. Indirect assessment of maternal condition may provide supporting evidence for long-lasting maternal effects. Abiotic variables (e.g., weather), for example, are strongly correlated with female condition [29], [30], [31] and general population performance [32], [33], [34], [35] in several ungulate species (e.g. white-tailed deer, red deer, and mule deer [*Odocoileus hemionus*], and elk [*Cervus canadensis*]). Additionally, climate factors are known to influence survival probability in mule deer and elk [36], [37], [38] and probability of survival is linked to body condition [39], [40]. Changes in forage availability and quality (excellent predictors of condition) with climate variation provide further support for the link between climate and condition [41], [42]. Thus, evaluation of these abiotic variables during gestation offers an indirect measure of maternal condition of the entire population – a variable that is otherwise difficult to discern without capturing individuals. While a suite of life history characters might be evaluated in relation to maternal condition, traits that are sexually selected (and therefore have the potential to influence reproductive success) are often most significant. Specifically, antler size is a sexually selected character that may be correlated with the lifetime reproductive success of ungulates [43], [44], [45]. While this relationship has not been rigorously demonstrated for all cervids, there is supporting evidence for a few species. For example, male mule deer with larger antlers are more likely to be socially dominant [46]. Similarly, American elk are closely related and have a similar life history to red deer (once considered the same species) where antler size is significantly correlated with lifetime reproductive success (r = 0.71) [43]. Population-level investigation of the relationship between an indirect measure of maternal condition and a sexually selected character (weather and antler size, respectively) should inform our understanding of maternal effects.

Our objective was to use indirect measures to determine if maternal condition has long-lasting influences on a sexually selected character of male cervids. More specifically, we examined the relative influence of climate variables during year of birth (a surrogate for maternal condition) on antler size. If climate variables from the year of birth have an effect on adult antler size at time of harvest, then maternal effects are long-lasting for these two species and are not entirely masked by environmental variables acting on offspring in the years following dependence. Conversely, if climate variables from year-of-birth do not affect antler size at time of harvest, then we would conclude that maternal effects do not exist or are masked by environmental variables in the years between the period of dependence and adulthood for long-lived species.

## Methods

### Data Collection

**Figure 1:**
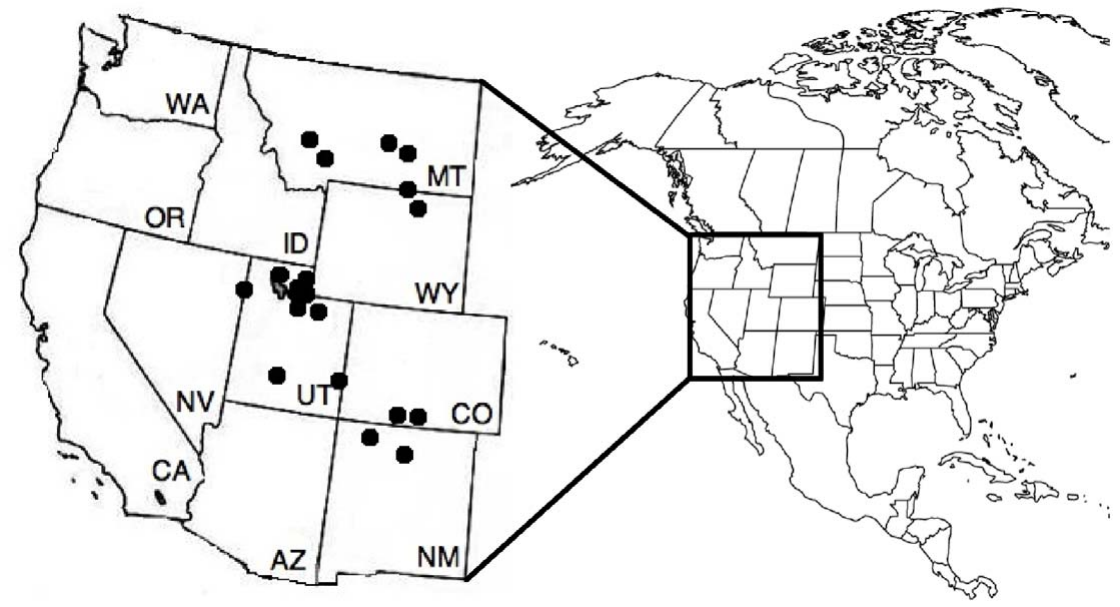
Site Locations. Map of North America with an inset of study sites (filled circles) in the western United States.

We collected data on annual climatic conditions during the year of birth of offspring (here considered a good proxy of maternal condition during gestation and lactation) and male antler size from two species, the American elk and the mule deer, across 20 free-range sites in the Intermountain United States. Sites were located in Colorado, Montana, Nevada, New Mexico, Utah, and Wyoming (Figure 1). These sites were privately or tribal-owned landholdings on which mule deer and/or elk were managed for sport harvest. We only collected data from hunter-harvested individuals, and had no part ourselves in handling or harvesting live individuals. We confirm that permission was granted by land-owners to those that harvested the individuals from which our data were collected in accordance with applicable state laws. We recorded antler size for mule deer and elk harvested between 1981 and 2010. Within this interval, the period of data collection at individual sites varied (Table 1). We obtained estimates of antler size by measuring the several antler characteristics quantified by the Boone and Crockett scoring system [47], an index of antler size accounting for length, mass, and spread of antlers.

**Table 1:** Sample distribution.

Mule Deer			Elk		
Site	# of individuals	Years of Data	Site	# of individuals	Years of Data
1	2670	29	10	191	11
2	103	7	16	14	1
3	84	9	17	57	12
4	197	11	14	28	4
5	175	9	3	52	8
6	324	16	18	91	4
7	608	13	18	460	14
8	91	11	20	2262	14
9	111	11	6	714	16
10	58	10	1	1053	28
11	23	4	4	139	13
12	10	4	13	31	5
13	7	2	12	16	6
14	45	5	11	34	4
15	42	9	8	153	12
TOTAL	4548		TOTAL	5295	

Description of data for mule deer (*Odocoileus hemionus*) and elk (*Cervus canadensis*) collected from 20 locations in western North America between 1981 and 2010.

We removed teeth from individuals following harvest and determined age by counting annuli in cementum [48]. This method of aging has previously been employed for our study species with a high degree of accuracy (97% for elk and 93% for mule deer) [49]. Based on this analysis, individuals were separated into cohorts ranging from 1.5–21.5 years old. Because younger individuals generally grow much larger antlers in subsequent years, while the antler size of older individuals changes very little in comparison, the influence of age on both elk and mule deer antler growth is a non-linear process between years [50]. To account for this variation among ages, we used age and age-squared in all models of antler size [45]. Furthermore, age data allowed us to account for age-based variation in antler size but also determine a year-of-birth for each individual harvested.

We paired each sample site with a national climatic data center (NCDC) weather station. We selected weather stations based on the station’s proximity and elevation relative to the site where sampling occurred. We chose the nearest station to the site if that station had collected data across all years data were available from the associated site. If the nearest station lacked data for some of the years, we selected the nearest station with similar temperature, precipitation, and snowfall averages to the station with insufficient data. If two stations were similar distances from the site, we selected the station nearer in elevation.

We obtained both winter and spring/summer weather data for each site from its corresponding NCDC weather station. Weather variables included the average temperature, precipitation, and snowfall for October through July for the year of harvest and the year of birth of each individual. In a few cases where these data were not available, we used the 40-year (1970–2010) average value for that site and variable to ensure that the missing data did not bias our analysis (imputation occurred for less than 1.5% of climate observations obtained from NCDC weather stations). From these data, we developed synthetic climate variables to minimize the number of parameters in our models. We determined a monthly z-score [51], [52], [53] by subtracting the 40-year average value for a given month from the observation for each individual (birth and harvest years), divided by the standard deviation of the 40-year average. We calculated separate z-scores for each of the three weather variables of interest (mean monthly temperature, total monthly precipitation, and total monthly snowfall). We then summed the z-scores for October – December, January – March, and April – July to create 3 synthetic parameters for each variable type (temperature, precipitation, and snowfall) and year of interest (birth or harvest). These synthetic variables were respectively Z-Early winter, Z-Late Winter, and Z-Summer. Our analysis therefore included nine year-of-birth (e.g. YOB.Ztemp.earlywinter, YOB.Ztemp.latewinter, and YOB,Ztemp.summer) and nine year-of-harvest effects for each individual.

### Statistical Analysis

We used model selection to determine relative support for biologically plausible, linear, mixed-effects models (separate analyses for elk and mule deer) of antler size where site was treated as a random effect. First, we modeled antler size as a function of year-of-birth or year-of-harvest influences (Tables 2 and 3). We judged relative support for these preliminary models based on the minimization of Akaike’s Information Criterion (AIC) [54] and AIC weights (*w_i_*) [55], [56]. Second, we used highly ranked year-of-birth and year-of-harvest variables (<2 ΔAIC of top model) identified in the first step to help develop a list of 14 *a priori* models for both elk and mule deer. These models described hypotheses about variation in antler size as a function of location, age, and weather influences (year-of-harvest and year-of-birth; Tables 4 and 5). We structured models to test the relative contribution of several factors (e.g. age, site, year-of-harvest weather, year-of-birth weather) to variation in antler size. While some models excluded year-of-birth weather variables, several models included them, in addition to site (random effect), age and age squared. Prior to running these *a priori* models, we verified that collinearity among weather variables (i.e., −0.60< r <0.60) did not exist within individual models. We again judged relative support for these final models based on the minimization of Akaike’s Information Criterion [54] and AIC weights (*w_i_*) [55], [56]. We used models within 2 ΔAIC of the top model to assess the strength and direction associated with explanatory variables. To verify assumptions of normality and homoscedasticity in error structure for the top model, we visually inspected residual plots (e.g. histogram, scatterplot, and qqnorm of residuals). We used the lmer function within the lme4 package [57] of Program R (version 2.12.1) for all analyses [58].

**Table 2:** Preliminary hypotheses.

Model	Preliminary Hypothesis Description
1	Age, Age^2^, and Site (null)
2	Age, Age^2^, Site, and Early Winter
3	Age, Age^2^, Site, and Late Winter
4	Age, Age^2^, Site, and Summer
5	Age, Age^2^, Site, and Early & Late Winter
6	Age, Age^2^, Site, Early & Late Winter, and Summer

Preliminary models exploring which year-of-birth and year-of-harvest effects best predicted antler size for mule deer (*Odocoileus hemionus*) and elk (*Cervus canadensis*) in western North America during 1981–2010. Harvest site was included as a random variable in each of these models.

**Table 3:** Preliminary model selection results.

	*Mule Deer*						*Elk*					
	Modela,f	K^b^	AIC^c^	ΔAIC^d^	*w* e	deviance	Modela,f	K^b^	AIC^c^	ΔAIC^d^	*w* e	deviance
YOH Temp	6	1,6	39735	0	0.952	39712	3	1,4	50448	0	0.604	50434
	5	1,5	39741	6	0.047	39723	5	1,5	50449	1	0.366	50432
	3	1,4	39749	14	0.001	39735	6	1,6	50454	6	0.030	50432
	4	1,4	39774	39	0.000	39759	1	1,3	50474	26	0.000	50463
	1	1,3	39782	47	0.000	39772	2	1,4	50477	29	0.000	50463
	2	1,4	39782	47	0.000	39768	4	1,4	50478	30	0.000	50462
YOH Precip	3	1,4	39780	0	0.329	39765	1	1,3	50474	0	0.622	50463
	5	1,5	39780	0	0.329	39762	3	1,4	50476	2	0.229	50462
	1	1,3	39782	2	0.121	39772	4	1,4	50477	3	0.139	50463
	2	1,4	39782	2	0.121	39768	2	1,4	50483	9	0.007	50459
	6	1,6	39783	3	0.073	39761	5	1,5	50485	11	0.003	50458
	4	1,4	39785	5	0.027	39771	6	1,6	50489	13	0.001	50458
YOH Snow	2	1,4	39781	0	0.247	39767	2	1,4	50468	0	0.620	50455
	6	1,6	39781	0	0.247	39759	5	1,5	50470	2	0.228	50453
	1	1,3	39782	1	0.150	39772	6	1,6	50472	4	0.084	50453
	4	1,4	39782	1	0.150	39768	1	1,3	50474	6	0.031	50463
	5	1,5	39782	1	0.150	39764	3	1,4	50475	7	0.019	50461
	3	1,4	39784	3	0.055	39770	4	1,4	50475	7	0.019	50461
YOB Temp	3	1,4	39775	0	0.774	39760	4	1,4	50472	0	0.620	50457
	5	1,5	39778	3	0.173	39760	1	1,3	50474	2	0.228	50463
	1	1,3	39782	7	0.023	39772	2	1,4	50477	5	0.051	50463
	6	1,6	39782	7	0.023	39758	3	1,4	50477	5	0.051	50463
	2	1,4	39786	11	0.003	39772	6	1,6	50478	6	0.031	50457
	4	1,4	39786	11	0.003	39772	5	1,5	50479	7	0.019	50462
YOB Precip	2	1,4	39775	0	0.817	39760	4	1,4	50464	0	0.988	50459
	5	1,5	39779	4	0.111	39760	1	1,3	50474	10	0.007	50463
	6	1,6	39781	6	0.041	39758	2	1,4	50476	12	0.002	50463
	1	1,3	39782	7	0.025	39772	3	1,4	50477	13	0.001	50463
	3	1,4	39786	11	0.003	39772	5	1,5	50479	15	0.001	50462
	4	1,4	39786	11	0.003	39772	6	1,6	50479	15	0.001	50459
YOB Snow	2	1,4	39777	0	0.783	39763	1	1,3	50474	0	0.309	50463
	5	1,5	39781	4	0.106	39763	3	1,4	50474	0	0.309	50460
	1	1,3	39782	5	0.064	39772	2	1,4	50476	2	0.114	50462
	6	1,6	39784	7	0.024	39763	4	1,4	50476	2	0.114	50463
	4	1,4	39785	8	0.014	39772	5	1,5	50476	2	0.114	50459
	3	1,4	39786	9	0.009	39772	6	1,6	50478	4	0.042	50459

Preliminary models described the response of antler size to year-of-birth or year-of-harvest weather factors we investigated as potential contributors to the yearly extent of antler growth in mule deer (*Odocoileus hemionus*) and elk (*Cervus canadensis*). Data were collected from 27 sites across western North America during 1981–2010. Model descriptions are located in Tables 1 and 2.

a Model number.

b Number of model parameters (random, fixed).

c Akaike’s Information Criteria.

d AIC relative to the best fitting model.

e Akaike weight.

f Models were judged to include uninformative parameters based on little to no improvement in deviance and fact that model differed from the top by a single variable.

**Table 4:** *A priori* hypotheses describing the response of mule deer (*Odocoileus hemionus*) antler size to selected explanatory variables.

Model	Hypothesis Description
1	Antler size by Age+Age^2^+Site (null)
2	Antler size by Age+Age^2^+Site+YOH temp early, late, and summer
3	Antler size by Age+Age^2^+Site+YOH prec early and late
4	Antler size by Age+Age^2^+Site+YOH snow early, late, and summer
5	Antler size by Age+Age^2^+Site+YOH temp early, late, and summer+YOH prec early and late
6	Antler size by Age+Age^2^+Site+YOH temp early, late, and summer+YOH snow early, late, and summer
7	Antler size by Age+Age^2^+Site+YOH early and late+YOH prec early and late
8	Antler size by Age+Age^2^+Site+YOH early and late+YOH snow early and late
9	Antler size by Age+Age^2^+Site+YOH temp early, late, and summer+YOH snow early, late, and summer+YOB temp late
10	Antler size by Age+Age^2^+Site+YOH temp early, late, and summer+YOH snow early, late, and summer+YOB temp late+YOB prec early
11	Antler size by Age+Age^2^+Site+YOH temp early, late, and summer+YOH snow early, late, and summer+YOB temp late+YOB prec early+YOB snow early and late
12	Antler size by Age+Age^2^+Site+YOH temp early and late+YOH snow early and late+YOB temp late
13	Antler size by Age+Age^2^+Site+YOH temp early and late+YOH snow early and late+YOB temp late+YOB prec early
14	Antler size by Age+Age^2^+Site+YOH temp early and late+YOH snow early and late+YOB temp late+YOB prec early+YOB snow early and late

Potential explanatory variables included site, age, and climate for year-of-birth and year-of-harvest on antler size of mule deer (*Odocoileus hemionus*) in western North America during 1981–2010. Harvest site was included as a random variable in each of these models.

**Table 5:** *A priori* hypotheses describing the response of elk (*Cervus canadensis*) antler size to selected explanatory variables.

Model	Hypothesis Description
1	Antler size by Age+Age^2^+Site*
2	Antler size by Age+Age^2^+Site+YOH temp early and late
3	Antler size by Age+Age^2^+Site+YOH prec late
4	Antler size by Age+Age^2^+Site+YOH snow early and late
5	Antler size by Age+Age^2^+Site+YOH temp early and late+YOH prec late
6	Antler size by Age+Age^2^+Site+YOH temp early and late+YOH snow early and late
7	Antler size by Age+Age^2^+Site+YOH prec late+YOH snow early
8	Antler size by Age+Age^2^+Site +YOH early and late+YOH prec late+YOH snow early
9	Antler size by Age+Age^2^+Site+YOH temp early and late+YOB temp summer
10	Antler size by Age+Age^2^+Site+YOH temp early and late+YOB temp summer+YOB snow early and late
11	Antler size by Age+Age^2^+Site+YOH temp early and late+YOB temp summer+YOB prec summer+YOB snow early and late
12	Antler size by Age+Age^2^+Site+YOH temp early and late+YOH snow early and late+YOB temp summer
13	Antler size by Age+Age^2^+Site+YOH temp early and late+YOH snow early and late+YOB temp summer+YOB snow early and late
14	Antler size by Age+Age^2^+Site+YOH temp early and late+YOH snow early and late+YOB temp summer+YOB prec summer+YOB snow early and late

Potential explanatory variables included site, age, and climate for year-of-birth and year-of-harvest on antler size of elk (*Cervus Canadensis*) in western North America during 1981–2010. Harvest site was included as a random variable in each of these models.

## Results

### Mule Deer

**Figure 2:**
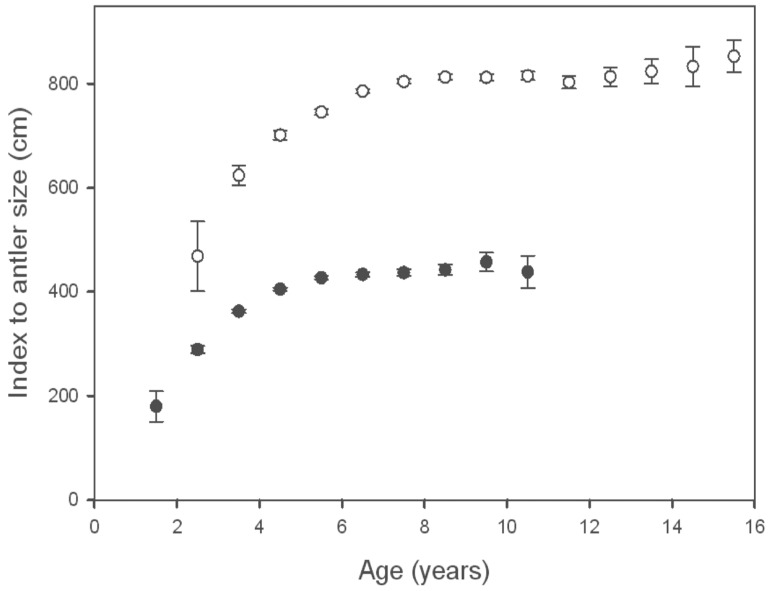
Antler Size and Age. Relationship between age and index of antler size (i.e., Boone and Crockett score in cm) for mule deer (*Odocoileus hemionus*; closed circles) and American elk (*Cervus canadensis*; open circles) collected from 20 areas of western North America during 1981–2010. Older age classes (11.5–12.5 for deer and 16.5–21.5 for elk) were not included in this figure because of small sample size.

**Figure 3:**
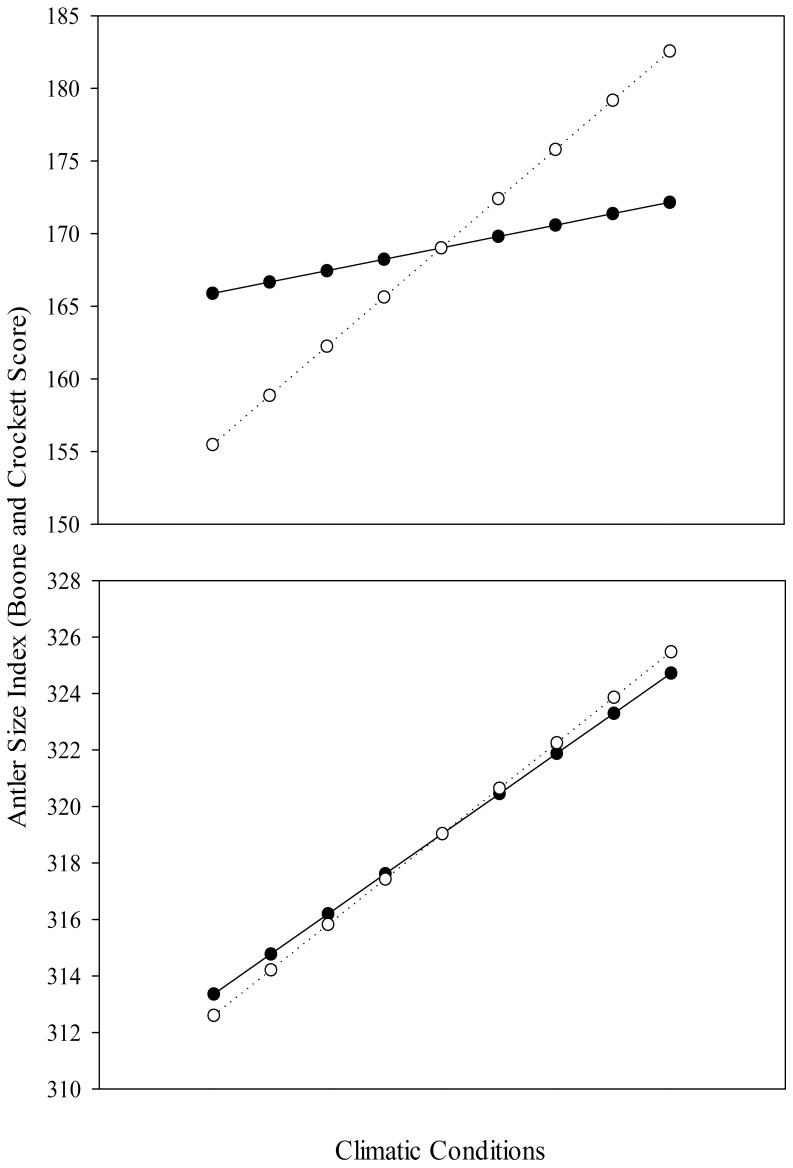
Effect Sizes of Climate Conditions. The predicted effect of climatic variables on antler size for mule deer (*Odocoileus hemionus;* A) and American elk (*Cervus canadensis;* B). Relative effects of climatic conditions during year-of-birth (solid lines) and year-of-harvest (dashed lines) over a continuum of favorable to unfavorable climatic variation. Data were collected from 20 areas in western North America during 1981–2010.

We collected age and antler measurements from 4,548 mule deer (Table 6). Results of model selection indicated a clear top model (no competing models <2 ΔAIC) accounting for 72.9% of the total AIC weight (Table 7). Average antler size increased rapidly from one age class to the next until 4.5 years, after which average antler size in subsequent years was relatively constant (Figure 2). Our most parsimonious model included year-of-birth effects. In addition to site, age, and several year-of-harvest effects, the top model included late-winter temperature and early-winter precipitation from the year-of-birth. We used the top model to assess strength and direction associated with explanatory variables from the year-of-birth (Table 8). Late-winter temperature (year-of-birth) was positively correlated with antler size, while early-winter precipitation (year-of-birth) was negatively correlated with antler size. Both of these variables indicate that mild winters prior to an individual’s birth positively influenced the antler size of that individual throughout its lifetime in our sample. The effect size associated with these two variables on antler size of mule deer was 8% from the most to least favorable climate conditions observed in our data set (Figure 3). However, the majority of individuals were born during more average years, and effect size was estimated at 4% across this continuum (cumulative monthly z score from -4 to 4). Climate conditions during the harvest year had a stronger influence as we estimated a 28% change in average antler size from the most to least favorable years observed and 18% across the range of values most commonly observed.

**Table 6:** Age and antler size description.

Mule Deer				Elk			
Age	SD	Mean	Sample Size	Age	SD	Mean	SampleSize
1.5	29.28	70.70	25	1.5			0
2.5	21.57	113.78	231	2.5	50.37	184.38	14
3.5	20.31	142.75	766	3.5	44.35	245.50	131
4.5	20.32	159.43	1361	4.5	35.20	275.93	412
5.5	20.99	168.02	1120	5.5	31.51	293.39	860
6.5	21.58	170.46	662	6.5	29.48	309.36	1022
7.5	23.04	171.94	284	7.5	29.06	316.58	966
8.5	21.15	173.99	117	8.5	30.55	319.85	735
9.5	25.59	179.94	49	9.5	29.66	319.71	515
10.5	29.18	172.42	22	10.5	28.19	320.90	276
11.5		197.00	1	11.5	33.55	315.94	195
12.5	21.70	161.66	4	12.5	36.04	320.16	95
				13.5	32.12	324.28	47
				14.5	33.93	327.99	20
				15.5	25.31	335.75	16
				16.5	15.11	347.31	13
				17.5	4.67	355.08	2
				18.5	26.29	322.68	4
				19.5	5.34	307.98	2
				20.5	35.36	310.42	3
				21.5		313.53	1

Description of antler size data for mule deer (*Odocoileus hemionus*) and elk (*Cervus canadensis*) collected from 20 locations in western North America between 1981 and 2010.

**Table 7:** Model selection results.

Species	Modela,f	K^b^	AIC^c^	ΔAIC^d^	*w* e	Deviance
*Mule Deer*	10	1,11	39709	0	0.729	39667
	11	1,13	39712	3	0.163	39667
	9	1,10	39713	4	0.099	39675
	6	1,9	39718	9	0.008	39685
	13	1,9	39722	13	0.001	39688
	12	1,8	39723	14	0.001	39693
	14	1,11	39725	16	0.000	39688
	8	1,7	39728	19	0.000	39702
	5	1,8	39733	24	0.000	39702
	2	1,6	39735	26	0.000	39712
	7	1,7	39743	34	0.000	39717
	3	1,5	39780	71	0.000	39762
	4	1,6	39781	72	0.000	39759
	1	1,3	39782	73	0.000	39772
*Elk*	10	1,8	50445	0	0.342	50417
	8	1,7	50447	2	0.126	50424
	12	1,8	50447	2	0.126	50421
	5	1,6	50448	3	0.076	50428
	9	1,6	50448	3	0.076	50427
	11	1,9	50448	3	0.076	50417
	14	1,11	50448	3	0.076	50412
	2	1,5	50449	4	0.046	50432
	6	1,7	50450	5	0.028	50427
	13	1,10	50450	5	0.028	50420
	4	1,5	50470	25	0.000	50453
	7	1,5	50471	26	0.000	50454
	1	1,3	50474	29	0.000	50463
	3	1,4	50476	31	0.000	50462

Models described the response of antler size to factors we investigated as potential contributors to the yearly extent of antler growth in mule deer (*Odocoileus hemionus*) and elk (*Cervus canadensis*). Data were collected from 27 sites across western North America during 1981–2010. Model descriptions are located in Tables 1 and 2.

a Model number.

b Number of model parameters (random, fixed).

c Akaike’s Information Criteria.

d AIC relative to the best fitting model.

e Akaike weight.

f Models were judged to include uninformative parameters based on little to no improvement in deviance and fact that model differed from the top by a single variable.

**Table 8:** Parameter estimates from fixed effects for the model that best accounted for antler size in mule deer and elk.

Species	Parameter	Estimate	SE	t value
*Mule Deer*	(Intercept)	62.62376	4.23263	14.8
	Age	32.45755	0.90237	35.97
	Age2	−2.27687	0.07797	−29.2
	YOH.Ztemp.earlywinter	−0.43601	0.17139	−2.54
	YOH.Ztemp.latewinter	1.11437	0.14774	7.54
	YOH.Ztemp.summer	0.3631	0.13726	2.65
	YOH.Zsnow.earlywinter	0.52515	0.17783	2.95
	YOH.Zsnow.latewinter	0.65988	0.17573	3.76
	YOH.Zsnow.summer	−0.28947	0.22433	−1.29
	YOB.Ztemp.latewinter	0.34342	0.12844	2.67
	YOB.Zprec.earlywinter	−0.44078	0.15801	−2.79
*Elk*	(Intercept)	191.59025	6.27292	30.54
	Age	25.783	0.8034	32.09
	Age2	−1.17196	0.04547	−25.77
	YOH.Ztemp.earlywinter	−0.34575	0.23946	−1.44
	YOH.Ztemp.latewinter	1.26332	0.2212	5.71
	YOB.Ztemp.summer	0.44898	0.15394	2.92
	YOB.Zsnow.earlywinter	0.38456	0.23536	1.63
	YOB.Zsnow.latewinter	0.58656	0.21888	2.68

Parameter estimates from fixed effects in the top model of mule deer (*Odocoileus hemionus;* top half) and American elk (*Cervus canadensis;* bottom half) antler size as a function of age and environmental conditions. Data were collected from 27 sites across western North America during 1981–2010.

### Elk

We collected age and antler measurements from 5,295 elk (Table 6). Results of model selection indicated that competing models (<2 ΔAIC) accounted for 59.4% of the total AIC weight (Table 7). Average antler size increased rapidly from one age class to the next until 6.5 years, after which average antler size in subsequent years was relatively constant (Figure 2). With the exception of one model (12.6% of AIC weight), all competing models included year-of-birth effects. In addition to age, harvest location, and year of harvest weather variables, our top model(s) included summer temperature and snowfall during both early and late winter from the year-of-birth. Because both year-of-birth and year-of-harvest weather variables were included in the top model(s), and were significant correlates of antler size, we concluded that maternal condition influenced a sexually selected trait of offspring in our sample. We used the top model to assess strength and direction associated with explanatory variables from the year-of-birth (Table 8). Our analysis indicated that increased summer (April – July) temperature during the year-of-birth was associated with greater antler size later in life. Additionally, increased snowfall during the year-of-birth was correlated with increased antler size throughout life. The effect size of maternal condition on antler size of American elk was 7.4% from the most to least favorable climate conditions that females experienced during the year-of-birth (Figure 3). However, the majority of individuals were born during more average years, and the effect size was 3.7% across this continuum (cumulative z score from −4 to 4). Effect size of climate conditions during the harvest year on the antler size of American elk was similar (8.4% across full range of observed conditions; 4.2% across commonly observed values) to that found during the birth year.

## Discussion

Our results indicate that year-of-birth environmental conditions influence the adult antler size at time of harvest for both elk and mule deer. Because environmental conditions (particularly during winter) often dictate an individual’s phenotypic quality (body mass, condition, etc.) [29], [30], [31] we can use weather data to make assumptions about a female’s ability or willingness to invest in her offspring, and thereby transmit a maternal effect [11], [12]. We therefore determined that maternal effects are present and impacting a life-history character in both elk and mule deer. Because the majority of our samples were harvested during their reproductive prime, we concluded that the observed maternal effects extended into adulthood and sexual maturity. Additionally, antler size is correlated to social dominance and potential reproductive success [43], [45], [46], [59], and we can therefore make some inference about the impacts of observed maternal effects on population dynamics.

Using climate indices as an indicator of maternal condition results in a proxy for average maternal condition across a population. Using this surrogate (inter-annual climate variation) limits our ability to detect individual variation (link individual males to individual females) and make inference about the effects of varying habitat quality, health of individual females, or other causes of varying female condition. However, based on current understanding of abiotic factors that influence condition, all individuals in a population should, on average, be either negatively or positively affected by climatic variation, albeit to different extents. Therefore, inter-annual variation in climate should be an acceptable surrogate for condition. Further, there was a significant effect of our proxy even though we could not account for variation among individual females (likely decreasing the power of our analysis). We recognize that through selective harvest our samples may potentially be biased toward individuals that grow larger antlers earlier in life. However, this potential bias should be consistent among locations and across the study period, and therefore unlikely to influence our conclusions.

As our data were collected on a broad temporal and spatial scale, we conclude that our results are applicable to elk and mule deer populations across the western United States. It is important however to consider the drastically different climates found in this region when interpreting results. For example, our analysis of elk antler size indicates that increased snowfall (October – July) during the year of birth is positively correlated with adult antler size. While this seems counter-intuitive for many western climates, water is likely the limiting factor in the desert systems of the southwest [60], [61] where nearly 60% of the elk included in our analysis were harvested. We concluded that parturient females from these sites were in better condition during years with any form of increased moisture. Additionally, because elk are larger and heavier than mule deer, they are likely more able to cope with severe weather conditions in climates where this is the limiting factor. The vast majority of our mule deer were harvested in northern systems where severe winters are likely a limiting environmental factor [36], [62], [63]. It is no surprise then that mild winters prior to an individual’s birth are associated with greater antler size during adulthood. Female mule deer from northern climates are likely in better condition during and following mild winters, leaving more energy after growth and maintenance functions for reproduction [29], [64]. In either case (elk or mule deer) the observed, significant correlations between weather and antler size would likely result in the transmission of a maternal effect (negative or positive dependent on the conditions and climate).

After detecting a maternally affected trait, the next logical step is to examine its evolutionary or biological consequences. Variance attributed to maternal effects (e.g. differential offspring size) may disappear during development through compensatory growth [65], thereby minimizing evolutionary consequences. For example, Dale et al. [66] examined the mass of reindeer cohorts at birth, and several times over the following 14 months, finding variation that existed at birth did not endure through their study, thereby limiting the effects of any observed variation. Conversely, variation that persists into adulthood and affects reproductive success can have a broad range of impacts. For example, if ecologists are concerned with investment in sexually selected characters or other life-history traits, the need for favorable conditions or nutrition during gestation is evident [14]. The sex ratio of offspring may also be impacted by the observed persistence of maternal effects. The Trivers and Willard Hypothesis [67] proposes that sex ratios of offspring in polygynous species should be biased and dependent upon maternal condition during gestation. This hypothesis is dependent on the assumptions that maternal effects on offspring are long-lasting and that male reproductive success is more variable than that of females [67]. While we cannot speak to differential reproductive success between males and females, we can support the assumption that maternal effects are long-lasting, at least for antler size. These and other impacts of maternal effects emphasize the importance of considering inherited environmental effects when interpreting population dynamics of long-lived organisms [14].

In conjunction with support for the maternal effects hypothesis, our analysis indicates that year-of-harvest environmental conditions have a significant impact on the antler growth, and therefore reproductive success of elk and mule deer. This is contradictory to recent findings by Vanpe et al. [68] that environmental conditions have no significant impact on antler growth of roe deer (*Capreolus capreolus*). Among other things, this discrepancy may be the result of location, sample size, or the species of ungulate in question. Vanpe et al. [68] proposed that this lack of influence might have resulted from a lack of climatic variation at their study locations during the study period. This explanation seems likely, as previous studies of ungulates have reported significant environmental influences on antler size. For example, Smith [69] reported that March and April temperatures accounted for 91% of the variation in antler size of elk for that year among similarly aged individuals (5.5–8.5 years). Similarily, Kruuk et al. [45] reported significant increases in antler mass correlated with summer rainfall (200 mm increase in precipitation was correlated with an average increase in antler mass of 23 g). We conclude that a lack of climatic variation may result in male ungulates with uniform antler size within a population relative to populations from areas with highly variable climates.

Our results do not support the assertion that environmental factors might mask any maternal influence on the reproductive success of male offspring [21]. Moreover, it appears that maternal effects on antler size are similar to the effects of environmental conditions from the current year–at least for elk and to a lesser degree for mule deer. The idea that maternal effects are potentially masked by environmental conditions for long-lived species has been argued because males disperse from their natal area and are exposed to the environmental conditions in their subsequent adult habitat for a few to several years prior to the potential opportunity for reproductive success [21]. It is likely that environmental conditions between the time of maternal dependence and adulthood influences antler size of adults and partially masks maternal effects. Nonetheless, our results indicate that maternal effects on antler size persist to adulthood–1.5 to 21.5 years later–even after exposure to many years of environmental forces during the interim.

These findings are consistent with those from previous examinations of ungulates; specifically that the effect of maternal condition lasts into adulthood and has the potential to influence reproductive success. The body mass of bighorn sheep ewes is associated with offspring mass at 4 months, which in turn is correlated with an individual’s subsequent adult (4 years) body mass [70]. Birth mass (often a correlate of maternal condition) of male white-tailed deer is correlated to subsequent adult (2.5 years) body mass [15]. Body mass of reindeer at 6 months of age is correlated to both mass at 18 months of age and body mass of their mothers [17]. These and similar studies use a measure of condition (body mass) that is correlated to reproductive success in many ungulate species [18], [71]; however, they fail to take these measures when individuals are likely to be dominant and reproductive. Other studies or meta-analyses have used longer time periods or older individuals [19], [21], [72], [73]. For example, mass of 3-week old bighorn sheep was correlated to mass and horn-length of adult males (5 years) and antler size of young red deer is correlated with antler size after 6 years [18], [74]. Similarly, our data were collected with a greater coverage of the reproductive lifetime of our study organisms and on a greater temporal and spatial scale.

Indirect measures have a history of being used to look for maternal effects on offspring [18], [19], [20]. Because maternal condition and the effects of maternal condition on offspring are difficult to quantify in free-ranging long-lived species, the usage of these and other indirect measures can provide insights that would otherwise be difficult to obtain. Our results indicate that maternal effects are present in our species and influence a sexually selected character. The large sample size and broad scale of our data make it unlikely that our analysis led to faulty conclusions. Additionally, our analysis includes samples that span the reproductive lifetimes of our study organisms, with the majority being taken from individuals in their reproductive prime (although this is an assumption, it is likely accurate because hunters generally attempt to harvest only the largest individuals on our sites). This was rarely accomplished in previous studies and is important when determining the consequences of an observed maternal effect. In this case, antler size is the maternally-influenced trait, indicating that the offspring of females in good condition will likely have larger antlers, a sexually selected trait.
